# Oxidative Stress and Cancer: Chemopreventive and Therapeutic Role of Triphala

**DOI:** 10.3390/antiox9010072

**Published:** 2020-01-13

**Authors:** Sahdeo Prasad, Sanjay K. Srivastava

**Affiliations:** Department of Immunotherapeutics and Biotechnology, and Center for Tumor Immunology and Targeted Cancer Therapy, Texas Tech University Health Sciences Center, Abilene, TX 79601, USA

**Keywords:** oxidative stress, cancer, antioxidant, triphala, ayurveda, chemoprevention and chemotherapy

## Abstract

Oxidative stress, caused by the overproduction of free radicals, leads to the development of many chronic diseases including cancer. Free radicals are known to damage cellular biomolecules like lipids, proteins, and DNA that results in activation of multiple signaling pathways, growth factors, transcription factors, kinases, inflammatory and cell cycle regulatory molecules. Antioxidants, which are classified as exogenous and endogenous, are responsible for the removal of free radicals and consequently the reduction in oxidative stress-mediated diseases. Diet and medicinal herbs are the major source of antioxidants. Triphala, which is a traditional Ayurvedic formulation that has been used for centuries, has been shown to have immense potential to boost antioxidant activity. It scavenges free radicals, restores antioxidant enzymes and non-enzyme levels, and decreases lipid peroxidation. In addition, Triphala is revered as a chemopreventive, chemotherapeutic, immunomodulatory, and radioprotective agent. Accumulated evidence has revealed that Triphala modulates multiple cell signaling pathways including, ERK, MAPK, NF-κB, Akt, c-Myc, VEGFR, mTOR, tubulin, p53, cyclin D1, anti-apoptotic and pro-apoptotic proteins. The present review focuses on the comprehensive appraisal of Triphala in oxidative stress and cancer.

## 1. Introduction

Cancer is a major health problem worldwide and the second leading cause of death. According to the American Cancer Society, 1,762,450 new cancer cases and 606,880 cancer deaths are projected to occur in the United States in 2019 [1]. However, from 2006 to 2015, it has been observed that the incidence rate of some cancers are either stable or have declined by approximately 2%. Moreover, the overall cancer death rate also dropped from 1991 to 2016 by a total of 27% [1]. This decline in cancer incidence and death is due to the significant drop in smoking and an increase in advances for early cancer detection and screening [2]. However, cancer is still a major health issue that burdens high care cost and causes physical and emotional difficulties to cancer patients. Besides preventive measures, several therapeutic modalities like surgery, chemotherapy and radiotherapy have been developed. These are very effective treatment measures but are very expensive, cause serious side effects, and subsequently may lead patients to the development of resistance to the therapy [3].

Several factors are associated with the causation of cancer. It may be caused by either external factors, internal factors or both. External factors include the consumption of tobacco and alcohol, exposure to hazardous chemicals, ionizing radiation, infectious organisms, and other lifestyle factors, whereas internal factors include inherited mutations, an imbalanced hormone level, and poor immune conditions [4]. These factors affect the incidence and mortality of cancer by modifying cellular systems of the organism. Internal factors such as hereditary mutation are not modifiable. Therefore, in order to control the incidence of cancer, external factors such as lifestyle and environmental factors need to be modified. This can be achieved through the cessation of smoking, minimal use of alcohol, increased consumption of fruits, vegetables and whole grains, physical activity, avoidance of direct exposure to sunlight, minimal red meat consumption, proper vaccinations, and routine screening. It has been shown that adopting changes in lifestyle can reduce over 90% of cancer incidence [5].

## 2. Oxidative Stress and Cancer

Free radicals, which are reactive oxygen species (ROS) and reactive nitrogen species (RNS), are constantly produced by biological systems. The antioxidants present in cells safely interact with the free radicals and neutralize them, thus establishing balance in the body. Oxidative stress occurs when there is an imbalance between the generation of free radicals and antioxidant defenses [6]. Free radicals are highly reactive and unstable molecules produced naturally as a byproduct of metabolism (oxidative phosphorylation), or by exposure to environmental factors. ROS, which include superoxide anion (O_2_^−^), hydrogen peroxide (H_2_O_2_), and hydroxyl radicals (OH•), are produced by the mitochondrial respiratory chain during oxidative metabolism through the one-electron reduction of molecular oxygen (O_2_) [7]. It is known that complexes I and II of mitochondria produce ROS only into the matrix, while complex III produces ROS on both sides of the mitochondrial inner membrane [8,9]. However, RNS which includes a nitric oxide radical (NO•), peroxynitrite (ONOO^−^), and a nitrogen dioxide radical (NO_2_•), are produced via the enzymatic activity of inducible nitric oxide synthase 2 (NOS2) and NADPH oxidase [10,11]. 

Oxidative stress is a crucial factor in the development of chronic diseases including cancer. Low levels of free radicals are implicated in many fundamental cellular processes such as immune defense, cellular proliferation and differentiation, activation of important signaling pathways and against pathogens. However, chronic and excessive amounts of ROS/RNS induce oxidative stress and cause deleterious effects to the cells [12]. They can induce oxidative damage to genetic materials, lipids, and proteins and further carcinogenesis and tumor progression. Increased oxidative stress also results in dysregulation of various cellular processes through modulation of signaling molecules, production of antioxidant enzymes and non-enzymes, cell growth, and chronic inflammation, which play major roles in the incidence of chronic diseases such as cancer [6]. 

Antioxidant systems are thus required to counteract oxidative stress and overcome cellular damage for the prevention of oxidative stress-mediated diseases like cancer. The cellular antioxidants are regulated by the transcription factor nuclear factor erythroid 2-related factor 2 (NRF2). Under an unstressed cellular condition NRF2 remains inactive by forming a complex with KEAP1 in the cytoplasm. NRF2 undergoes ubiquitination and further proteasomal degradation by KEAP1 through the Cullin 3 (CUL3) based E3 ligase [13]. However, disruption in binding of NRF2 to KEAP1 leads to the nuclear translocation of NRF2, where it regulates the basal and inducible expression of several genes that contain antioxidant response elements (AREs) [14,15]. NRF2 not only regulates redox homeostasis [16] but also the other physiology of cells [15]. NRF2 is reported to prevent chemical and radiation-induced carcinogenesis by quenching ROS or managing oxidative damage. However, since the last decade, a ‘dark side’ of NRF2 has also been described [17]. Some studies have shown that in cancer cells NRF2 activation promotes cancer progression [18,19], metastasis [20], and causes resistance to therapeutic agents [21]. These studies explain that the activation of NRF2 prevents carcinogenesis but may facilitate tumor growth and metastasis in cancer cells. Thus, activation of NRF2 is beneficial for the prevention of carcinogenesis but may not be beneficial for cancer treatment.

The antioxidants can be produced by cells endogenously or can be supplied to the cells through food and/or supplements exogenously [22]. Because of the limitation in endogenous production of antioxidant by cells, exogenous supplement of antioxidants can satisfy the requirement and thereby reduce oxidative stress-mediated cellular damage and carcinogenesis. Plant products are one of the major source of antioxidants, which have little or mild toxicity, are abundantly available, and have high efficacy and cost effectiveness [6,23,24,25]. Traditional medicines, which utilize a variety of medicinal plants, are the inherent source of antioxidants. Because of their disease curing capability, traditional medicines have been used for centuries against a variety of ailments [26]. Like other traditional medicines, Ayurvedic medicines consist of a single constituent or a mixture of different constituents of one or multiple medicinal plants. Ayurveda is an ancient Indian medical system and considered as one of the world’s oldest holistic healing systems, which is thought to have been developed more than 5000 years ago in India [26,27].

**Figure 1:**
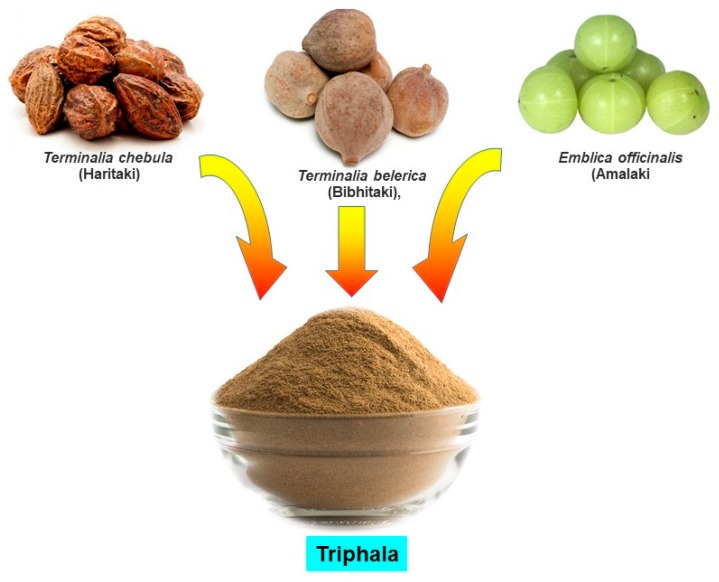
Constituents of Triphala.

Accumulated evidence suggests that Ayurvedic medicines exhibit antioxidant properties by neutralizing free radicals, quenching ROS, and lowering peroxides [6,28,29]. In a study, Ayurvedic medicine *Jeevaneeya Rasayana* (an ayurvedic polyherbal formulation) was found to increase the activities of antioxidant enzymes and the level of glutathione content in arthritic rats. This formulation also decreased the concentration of C-reactive protein, thiobarbituric acid reactive substance, and ceruloplasmin in arthritic rats [30]. Moreover, using an arthritic rat model, Ratheesh et al. [31] also found that the Ayurvedic formulation *Kerabala* increased antioxidant enzymes such as superoxide dismutase (SOD), catalase (CAT), glutathione peroxidase (GPx), and decreased the lipid peroxidation product. Another Ayurvedic formulation, *Amalakayas Rasayana*, also showed antioxidant activity by scavenging free radicals [32]. Mathew et al. [33] analyzed antioxidant activity of many Ayurvedic plant extracts and found all extracts had positive DPPH (2,2-diphenyl-1-picryl-hydrazyl-hydrate) free radical scavenging activity. These studies indicate that Ayurvedic plants or formulations have antioxidant and free radical scavenging activity that may explain its effect and justify its use as a medicine against oxidative stress-associated diseases such as cancer. A variety of Ayurvedic formulations have been described in Ayurveda, however in this article, we discuss an increasingly popular Ayurvedic formulation, Triphala (Figure 1).

## 3. Triphala: A Formulation of Three Fruits

**Figure 2:**
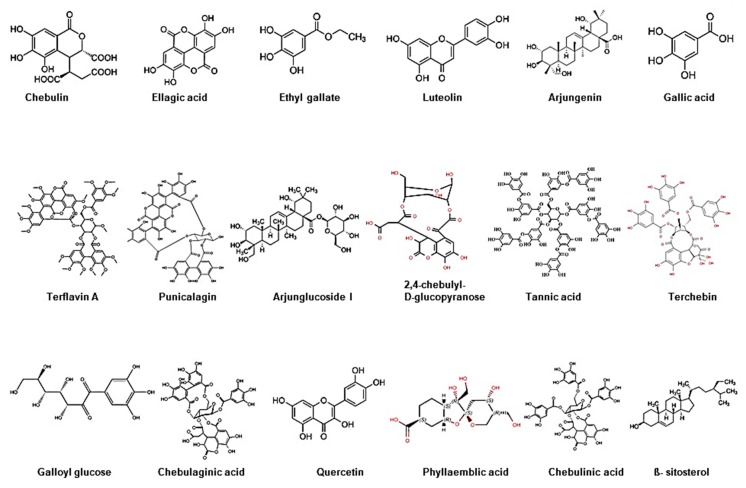
Chemical structure of bioactive components of Triphala.

Triphala, as the name indicates in the Sanskrit language (tri = three and phala = fruits), is a herbal formulation consisting of the dried powdered fruits of three plants, *Terminalia chebula* (Haritaki), *Terminalia belerica* (Bibhitaki), and *Phyllanthus emblica* or *Emblica officinalis* (Amalaki or the Indian gooseberry) (Figure 1). Although the Triphala formulation generally consists of equal proportions of fruits from these plants, a modified formulation consisting of 1:2:4 parts of *T. chebula*, *T. belerica*, and *E. officinalis* are also used [34]. Chemical analysis of *T. chebula* extract shows that it contains many biologically active constituents like chebulin, ellagic acid, 2,4-chebulyl-d-glucopyranose, arjunglucoside I, arjungenin, chebulinic acid, gallic acid, ethyl gallate, punicalagin, terflavin A, terchebin, luteolin and tannic acid. However, the main chemical constituents of *T. bellerica* are tannins that mainly include β-sitosterol, gallic acid, ellagic acid, ethyl gallate, galloyl glucose and chebulaginic acid [35]. The fruit of *P. emblica* has been shown to be rich in quercetin, phyllaemblic compounds, gallic acid, tannins, flavonoids, pectin, and vitamin C [36] (Figure 2).

A comparative study on *T. chebula*, *T. belerica* and *E. officinalis* (components of Triphala) have shown that they exhibit potent antioxidant activities. In a study, total antioxidant capacity was measured and results indicated that the *T. chebula* extract had a higher (4.52 ± 0.12) antioxidant capacity compared to *T. belerica* (1.01 ± 0.03) and *E. officinalis* (4.10 ± 0.17). The DPPH scavenging activity was found in the order of *T. chebula, T. belerica* and *E. officinalis* (1.73 ± 0.07 μg/mL, 1.45 ± 0.02 μg/mL & 1.43 ± 0.03 μg/mL). However, *T. chebula, T. belerica* and *E. officinalis* extracts showed a moderate effect on the scavenging singlet oxygen species with IC50 values of 424.50 ± 24.70 μg/mL, 233.12 ± 48.68 μg/mL and 490.42 ± 159.59 μg/mL, respectively [37]. These studies indicate that extracts of these fruits exhibit their antioxidative properties in the order of *T. chebula* > *E. officinalis* > *T. belerica*, which follow the order of their flavonoid contents [37].

Total phenolic, flavonoid and tannin contents were also analyzed in these fruits. The total phenolic content of *T. chebula* fruit extracts were varied from 867.2 to 1041.8 mg gallic acid/gm extract with the highest concentration of phenolic compounds in water extract followed by methanol and ethanol extracts. However, total triterpenoid content of the three extracts varied widely from 0.8 to 4.2 mg ursolic acid/gm extract with the lowest total triterpenoid content in water extract, whereas the methanol extract provided the highest triterpenoid content. The total tannin content of the three extracts varied from 33.9 to 40.3%/mg extract. The highest total tannin content was detected in the water extract followed by methanol and ethanol extracts [38]. However, *T. bellerica* fruits have shown flavonoids in ethanol and chloroform extracts but not in methanol extract. Additionally, triterpenoids and tannins were reported only in the ethanol extract [39]. The total phenolic content in *E. officinalis* has been reported to range from 188.8–237.0 mg gallic acid/gm. Nonetheless, the total flavonoid content ranged from 6.4–20.1 mg rutin/gm, whereas total tannin content ranged from 375.2–642.8 mg tannin/gm [40]. These studies indicate that *T. chebula* fruit extracts contain the highest amount of phytochemicals followed by *E. officinalis* and *T. chebula* fruits. Hazra et al. [37] also demonstrated that *T. chebula* fruits had the highest flavonoid content followed by *E. officinalis* and *T. belerica*. However, in the case of phenolic content, *E. officinalis* fruits had the highest, thereafter *T. belerica* and *T. chebula*. The variation in these phytochemical contents may depend on the geographical origin of these plants.

In Ayurveda, Tridosha defines three fundamental energies or principles (vata, pitta, and kapha) that govern the function of our bodies at the physical and emotional level [41]. Triphala is considered as a tridoshic rasayan that has the ability to balance and rejuvenate Tridosha, as well as promote health, immunity and longevity [42]. In Ayurvedic practice, Triphala is frequently used to treat digestion problems, poor food assimilation, constipation, and gastric acidity. Besides these, it is used in the treatment of many other diseases such as asthma, anemia, jaundice, fever cough, chronic ulcers, leucorrhoea, and pyorrhea. It is also recommended for use in the treatment of cardiovascular disorders, ophthalmic problems, liver dysfunction, inflammation, infection, obesity, anaemia, and fatigue [43]. Most people practicing Ayurvedic medicine consume Triphala as a ‘health tonic’. Triphala improves blood circulation, reduces myocardial necrosis and serum cholesterol levels, and strengthens capillaries, which indicates its cardiotonic effects [44]. Recent studies showed that it had antioxidant, anti-inflammatory, antiaging, anti-mutagenic, anti-clastogenic and anticancer effects. Herewith an attempt was made to summarize the antioxidant and cancer preventive and therapeutic aspects of Triphala (Table 1).

## 4. Antioxidant Effects of Triphala

**Figure 3:**
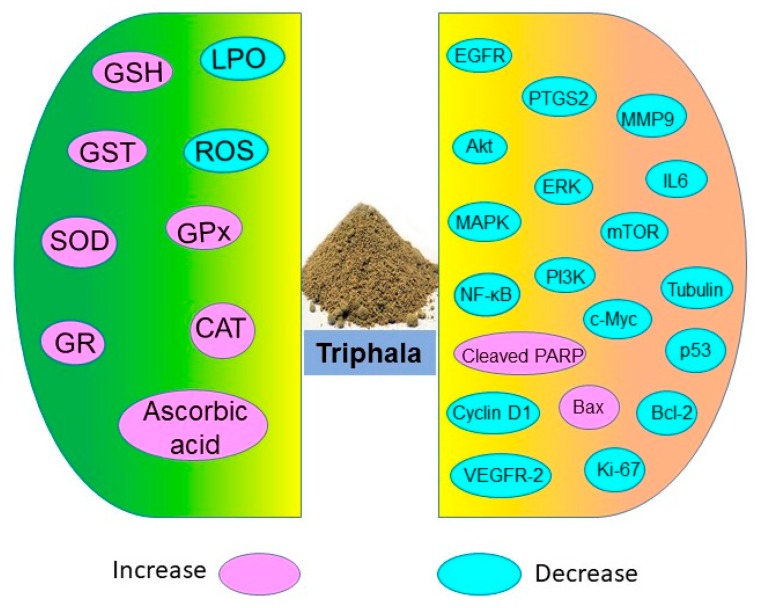
Antioxidative and chemoprotective molecules targeted by Triphala.

Triphala holds potential in restoring antioxidant levels and decreasing lipid peroxidation as shown in numerous in vitro, in vivo, and human studies (Figure 3). These antioxidant properties of Triphala are associated with the presence of polyphenols, vitamin C, and flavonoids. The active constituents of Triphala quench the ROS levels and reduce oxidative stress.

### 4.1. In Vitro Studies

Numerous in vitro studies have shown that Triphala has high antioxidant potential. In a study, both aqueous and methanolic extracts of Triphala were examined for antioxidant activities and were found to quench free radicals and induce SOD and CAT antioxidant enzymes. Triphala extract exhibited satisfactory free radical-scavenging activity that was comparable with ascorbic acid [47]. In HeLa cells, Triphala efficiently eliminates ROS levels generated by X-radiation and bleomycin and thus protects from X-radiation and bleomycin-mediated DNA strand breaks [45]. In another study, Triphala extract also inhibited radiation-induced lipid peroxidation in rat liver microsomes. The extracts were found to possess the ability to scavenge free radicals such as DPPH and superoxide. It was also found that Triphala extract had the ability to prevent gamma-radiation-induced strand break formation in plasmid DNA [48]. 

Triphala extract also inhibits H_2_O_2_-induced RBC haemolysis, nitric oxide production and shows high reducing power activity. As H_2_O_2_ induces cellular damage, it has been shown that pretreatment with Triphala rescues the human dermal fibroblast from H_2_O_2_-induced damage, inhibits cellular senescence, and protects DNA from damage [49]. Triphala has been found to increase glutathione (GSH) and decrease malondialdehyde levels in enucleated rat lenses. It can also restore the activities of antioxidant enzymes such as SOD, CAT, GPx, glutathione reductase (GR), and glutathione-S-transferase (GST) and improves selenite-induced cataract [52].

### 4.2. In Vivo Studies

Triphala is found to be effective in reducing oxidative stress in animal models. In a study, pretreatment with two doses (150 mg/kg and 300 mg/kg) of Triphala in a colitis rat model, it restored the antioxidant enzymes SOD and CAT, and decreased the malondialdehyde levels in the distal colon of rats. Triphala further relieved the rats from colitis, which could be attributed to its antioxidant activity [67]. In complete Freund’s adjuvant-induced arthritic rat model, Triphala showed antioxidant properties. Administration of Triphala (100 mg/kg b wt, i.p.) restored the activities/levels of antioxidant (SOD ~75.6%, CAT ~62.7%, GPx ~55.8%, GST ~82.1%, and GSH ~72.7%), and decreased the lipid peroxidation in the paw tissues of arthritic rats [68]. A study on another monosodium urate crystal-induced arthritis model showed support for Triphala exhibiting antioxidant properties and decreasing inflammation. Oral treatment of Triphala (1 g/kg) inhibited paw volume and lipid peroxidation; however the antioxidant status was found to be increased in the plasma, liver, and spleen of monosodium urate crystal-induced mice when compared to control mice [69].

Triphala also exerts nephroprotective effects due to its antioxidant properties. In Wistar albino rats, bromobenzene treatment resulted in a decrease in the activities of antioxidant enzymes such as CAT, SOD, GST, and GPx as well as total reduced GSH in the kidney. Bromobenzene also increased lipid peroxidation in the kidney of animals. However, oral administration of two different doses (250 and 500 mg/kg) of Triphala in bromobenzene-treated rats restored antioxidant enzymes and decreased lipid peroxidation [50]. Thus, data indicates that Triphala has nephroprotective effects through its antioxidant nature. The antioxidative property of Triphala is also directed toward the protection of carcinogen-induced cellular damage. It has been shown to prevent 1,2-dimethylhydrazine dihydrochloride (DMH)-induced mouse liver damage by decreasing DMH-induced lipid peroxidation and increasing GSH and GST [51].

Administration of 25 mg/kg Triphala to the animals decreased nuclear cataract in the selenite-induced cataract model [52]. Sandhya et al. [46] have also shown that Triphala protects against radiation-induced oxidative damage in mice. They have found that 5 Gy radiation induces mortality in mice. However, oral treatment of Triphala (1 g/kg) reduced mortality by 60% in mice. This improvement was found to be associated with an increase in antioxidant enzymes such as SOD and protection from DNA damage in the intestine of mice exposed to irradiation. Triphala administration in animals also increased radiation tolerance, which was further mediated through its antioxidant activity and scavenging free radicals [70]. 

Noise-stress causes alterations in the antioxidant status and on the cell-mediated immune response. Thus, to determine the protective effect of Triphala, noise-stress (100 dB for 4 h/d/15 days) was employed on the rats and Triphala (1 g/kg/bw/48 days) was administered to the animals. Treatment with Triphala resulted in a decrease in noise-stress-induced lipid peroxidation and corticosterone level with concomitant increase of antioxidants in the plasma and tissues of rats. Thus, the study indicates that Triphala has preventive effects on noise-stress induced changes by increasing antioxidants as well as modulating the cell-mediated immune response in rats [53]. Besides these, equal (1:1:1) and unequal (1:2:4) formulations of Triphala have also been compared to determine their antioxidant and enteroprotective efficacy on methotrexate-induced small intestinal damage in rats. It has been observed that the unequal formulation of Triphala provides significantly more protection by restoring GSH than equal formulation of Triphala against methotrexate-induced damage in the rat intestine [34].

## 5. Prooxidant Nature of Triphala

Besides its antioxidant property, Triphala also exhibits prooxidant activity by inducing ROS production in cancer cells. Triphala has shown an insignificant level of ROS production in normal breast MCF-10F cells as well as in murine spleen and liver normal cells [55]. As increased levels of ROS in cancer cells causes lethality [71,72], the prooxidant nature of Triphala escalates the death of cancer cells and acts as an anticancer agent. In a study, it has been observed that Triphala inhibited proliferation and induced apoptosis in MCF-7 and T47D breast cancer cells through production of ROS. Further, it was observed that quenching ROS by antioxidants inhibited the anti-proliferative ability of Triphala suggesting its role in the induction of apoptosis through ROS production [56]. Triphala has also been shown to induce ROS generation in Capan-2 pancreatic cancer cells and further apoptosis. Triphala-induced ROS generation also led to phosphorylation of p53 and ERK in Capan-2 cells as pretreatment combined with the antioxidant N-acetylcysteine blocked Triphala-induced phosphorylation of these proteins [57]. Cancer cells are known to have high levels of ROS. Increasing ROS further crosses the threshold and forces the cell into apoptosis [72]. However, the level of ROS in normal cells is very low and not easy to raise to go over the threshold limit.

As a prooxidant, Triphala also produces a radiosensitizing action through oxidative damage, membrane alteration and damage to nucleic acids in various cancer cell lines. In tumor cell lines such as Ehrlich ascites (EAC), human cervical (HeLa), and breast (MCF-7) cells, treatment with Triphala induced a cytotoxic effect by initiating membrane oxidative damage and by triggering ROS generation by gamma radiation [73]. In contrast, Triphala showed protective effects against X-radiation and bleomycin in HeLa cells. However, it has also shown protective activity against ionizing radiation in mice [45].

## 6. Chemopreventive and Chemotherapeutic Effects

Extensive studies on Triphala have shown that it has preventive and therapeutic efficacy against malignancies such as breast, colon, pancreas, prostate, ovarian, cervical, endometrial, and lymphatic cancers as well as melanoma [58,60,61,63,74]. Although Triphala has been used for centuries against various ailments, recent in vitro, in vivo and human studies have demonstrated its safety and efficacy against multiple diseases including cancer. Experimental studies in the past decade have also shown that Triphala exhibits antineoplastic, radioprotective, and chemoprotective effects through modulation of multiple signaling molecules (Figure 3).

### 6.1. In Vitro Studies

Triphala has shown anticancer activities in various cancer cell lines. Using a cytotoxic assay, it was found that the aqueous extract of Triphala decreased the proliferation of breast and prostate cancer cells. Further chemical analysis showed that the extract was rich in polyphenol gallic acid, which was considered as a major factor in inducing cytotoxicity of cancer cells [63]. Sandhya et al. [55] also showed that the increasing concentrations of Triphala correspondingly decreased the viability of treated breast cancer MCF-7 cells. Besides cytotoxicity, Triphala treatment was found to induce apoptosis in MCF-7 and barcl-95 cells in vitro. Further mechanistic studies of Triphala on apoptosis and cytotoxicity were demonstrated by using single cell gel electrophoresis in breast cancer cells and found that it increased intracellular ROS and induced DNA damage, a characteristic of apoptosis. However, with similar concentrations of Triphala, it did not cause any cytotoxic effect or DNA damage on normal breast epithelial cells, MCF-10F, human peripheral blood mononuclear cells, mouse liver and spleen cells. This study indicated that Triphala was selectively cytotoxic to the cancer cells. 

Further studies revealed the crucial role of p53 in Triphala-mediated apoptosis in breast cancer cells. It was observed that MCF-7 cells with wild type p53 were more sensitive to Triphala than p53 negative T47D breast cancer cells. Triphala-induced ROS generation plays a major role in apoptosis, since the addition of antioxidants inhibits the anti-proliferative ability of Triphala [56]. In contrast, it has been reported that the methanol extract of Triphala suppresses the proliferation of colon cancer HCT116 cells and human colon cancer stem cells (HCCSCs) independent of p53 status. This extract also induced p53-independent apoptosis in HCCSCs as indicated by elevated levels of cleaved PARP. It further suppressed c-Myc and cyclin D1, and induced apoptosis through elevation of Bax/Bcl-2 ratio. In addition, Triphala extract inhibited HCCSCs colony formation, a measure of CSCs self-renewal ability [62]. It is worth noting that Triphala scavenges ROS in normal cells thereby preventing oxidative damage, whereas it increases the ROS level and causes lethality in cancer cells. 

Growth-inhibitory effects of Triphala were also evaluated in pancreatic cancer cells. It was observed that treatment with the aqueous extract of Triphala reduced the survival of pancreatic cancer Capan-2 cells. Triphala-mediated reduction in cell survival was correlated with the induction of apoptosis. Further it was shown that Triphala extract induced ROS production that led to phosphorylation of p53 and ERK in Capan-2 cells and then apoptosis, whereas antioxidant N-acetylcysteine (NAC) treatment blocked apoptosis [57]. In another study, Triphala inhibited the proliferation of multiple cancer cells such as HeLa (cervical adenocarcinoma), PANC-1 (pancreatic adenocarcinoma), and MDA-MB-231 (triple-negative breast carcinoma) cells and suppressed the clonogenicity of HeLa cells. The mechanism of the antiproliferative effect was mediated by disruption of secondary conformation of tubulin and inhibition of anilino naphthalene sulfonate binding to tubulin. Triphala acetylates cellular microtubules and stabilizes microtubule dynamics. In addition, Triphala interfered with the reassembly of microtubules. The microtubule interfering effects of Triphala lead to apoptotic cell death in cancer cells [61].

Triphala is also effective in suppressing gynecological cancer cell growth. Treatment with Triphala inhibited proliferation and induced apoptosis in SKOV-3, HeLa, and HEC-1B cells. The antiproliferative and proapoptotic activities were confirmed by cell cycle analysis and expression of Ki-67 protein. It was also found that Triphala decreased the expression of phospho-Akt, phospho-p44/42, and phospho-NF-κB p56 in these gynecological cancer cells, which indicated that MAPK/ERK, PI3K/Akt/mTOR, and NF-κB/p53 signaling pathways were the possible mechanism of Triphala-induced apoptosis [60]. Besides its anti-proliferative and apoptotic effects, Triphala suppressed cell migration of cancer cells in vitro thus indicating its anti-metastatic potential [58].

### 6.2. In Vivo Studies

Triphala also has cancer chemopreventive potential as shown in animal studies. In a study, Triphala (2.5%, supplemented in diet) significantly reduced benzo(a)pyrene [B(a)P] induced forestomach papillomagenesis in mice. It reduced tumor incidence by 77.77% in the short-term study and 66.66% in the long-term study. As it is a potent antioxidant, the chemopreventive effect of Triphala might be associated with an increased antioxidant status in animals [54]. Oral administration of Triphala (50–100 mg/kg) also suppresses the growth of Capan-2 pancreatic tumor-xenograft. It was found that reduction in tumor growth by Triphala in mice was due to increased apoptosis in the tumor cells, which was associated with increased activation of p53 and ERK [57]. Another study also revealed that tumor inhibitory effects of Triphala or its active constituents were through suppression of VEGF actions. Triphala and one of its active compounds, chebulinic acid, specifically inhibits VEGF-induced angiogenesis by suppressing VEGF receptor-2 (VEGFR-2) phosphorylation and thus reduces tumor growth and metastasis [75]. In a zebrafish xenograft model, administration of Triphala inhibited the growth and metastasis of transplanted gastric carcinoma cells. The antineoplastic effect of Triphala was analyzed by western blotting and results demonstrated that it inhibited phosphorylation of EGFR, Akt, and ERK [58].

## 7. Immunomodulatory Effect of Triphala

Triphala was shown to alter the immune system and act as an immunomodulatory agent. In a published study, the immunomodulatory activity of Triphala was assessed by testing the various functions of neutrophil-like adherence, phagocytosis and avidity index in albino rats. Upon Triphala administration, the avidity index was found to be increased in the animals. The neutrophil functions were also enhanced in the Triphala immunized group with a decrease in the corticosterone level [64]. Thus, Triphala appears to stimulate neutrophil functions in the immunized rats and prevent stress-induced suppression of neutrophil functions. Another study showed that the supplementation of Triphala prevented the noise-stress induced changes in the cell-mediated immune response in rats [53]. Immunostimulatory activity of Triphala was also evaluated in a phase I clinical study. Consumption of Triphala by healthy volunteers demonstrated significant immunostimulatory effects on cytotoxic T cells (CD3^−^ CD8^+^) and natural killer cells (CD16^+^ CD56^+^). However, Triphala did not change the cytokine level in volunteers [66]. The individual components of Triphala have also shown to exhibit immunomodulatory activity. The *T. chebula* fruit extract has illustrated an increase in spleen lymphocyte proliferation and enhanced the expression of cytokines such as IL-2, IL-10 and TNF-α in rats [76]. The methanolic extract of *T. bellerica* has affected the mouse immune system, specifically both the cellular and humoral immune response in vitro. This extract stimulated phagocytic activity and T-lymphocyte proliferation [77]. *E. officinalis* fruit extract exhibited immunostimulatory activity by its combined action on humoral and cell-mediated immune responses along with macrophages and phagocytes [78]. Thus, these studies indicate that Triphala and its three individual constituents have potential to stimulate immune systems.

## 8. Conclusions

**Figure 4:**
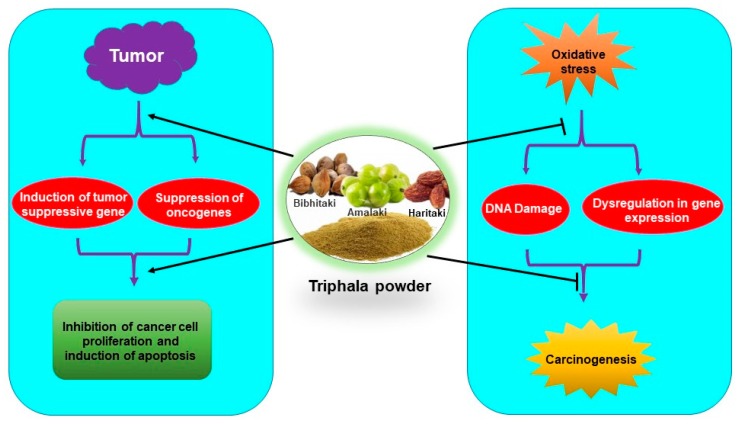
Antioxidative and chemopreventive/chemotherapeutic properties of Triphala.

Triphala has been used for centuries against various ailments in the Indian traditional medicine system. Studies in the recent past have indicated that Triphala has immense potential in the reduction of oxidative damage as well as in the prevention and treatment of cancer (Figure 4). Few studies indicated that antioxidants from dietary supplements may promote tumor growth and metastasis [79,80,81]. However, it is noteworthy that Triphala acts as an anticancer agent by exhibiting prooxidant effects in cancer cells. The dual nature of Triphala, acting as an antioxidant in normal cells and prooxidant in cancer cells, facilitates its function as both a chemopreventive and chemotherapeutic agent. Interestingly, Triphala has shown high efficacy and safety in humans as well as in experimental studies. However, most of the studies are done in animals and in vitro models. Clinical studies are required for its applicability as a chemopreventive, radioprotective, and chemotherapeutic agent. Three clinical trials on Triphala have been completed and another one is underway on different diseases like gut microbiome and skin (NCT03477825), gingivitis (NCT01898000), periodontal disease (NCT01900535) and stool microbiome and inflammation (NCT03907501). However, up to now, no clinical trial on Triphala in cancer has been done. Therefore, clinical studies to determine its efficacy in cancer patients are warranted. As 1:2:4 of Triphala has shown better enteroprotective effects over the conventional 1:1:1 combination, examining the effect of Triphala in different combination ratios is required with the hope that new formulations may exhibit better beneficial effects on oxidative stress-mediated chronic diseases.

## Figures and Tables

**Table 1:** Antioxidant and chemotherapeutic effect of Triphala.

Effects	Studies	References
ROS scavenging	Eliminates X-radiation-induced ROS generation in HeLa cells.	[45]
	Quenches γ-radiation-induced free radicals.	[46]
	Scavenges free radicals comparable with ascorbic acid.	[47]
	Scavenges free radicals such as DPPH and superoxide.	[48]
Antioxidant enzymes	Increases expression of SOD-2 in HDF or HaCaT skin cells.	[49]
	Restores CAT, SOD, GST, GPx and GSH in bromobenzene treated rat kidney.	[50]
	Prevents peroxidative damage by increasing GSH and GST and decreasing LPO in DMH treated mouse liver.	[51]
	Restores GSH, CAT, SOD, GPx, and GST in cataract mouse model.	[52]
	Restores GSH content and decreases LPO in MTX-induced small intestinal damage in rats.	[34]
	Prevents noise-stress induced decrease in SOD, CAT, GPx, ascorbic acid, and increase in LPO in plasma and thymus tissues.	[53]
	Inhibits γ-radiation-induced lipid peroxidation in rat liver microsomes.	[48]
Radioprotective	Prevents γ-radiation-induced DNA damage in HeLa cells.	[45]
	Prevents DNA damage in blood leukocytes and splenocytes of mice exposed with whole body γ-radiation.	[46]
Chemopreventive	Reduces B(a)P-induced forestomach papillomagenesis in mice at a dose of 2.5% and 5% in diet.	[54]
Prooxidant	Increases ROS level and induces apoptosis in breast cancer MCF-7 and barcl-95 cells.	[55]
	Induces ROS and inhibits proliferation in MCF 7 and T47D breast cancer cells.	[56]
	Induces apoptosis and phosphorylation of p53 and ERK through ROS generation in Capan-2 cancer cells.	[57]
Therapeutic	Decreases survival and induces apoptosis in Capan-2 pancreatic cells cancer with an IC50 of 50 µg/mL.	[57]
	Inhibits gastric cancer cell proliferation and suppresses cell migration in vitro.	[58]
	Exerts anti-proliferative, apoptotic and anti-migratory effects in colon cancer cells.	[59]
	Inhibits proliferation of gynecological cancers cell with IC50 values of 98.28–101.23 µg/mL against SKOV-3, HeLa, and HEC-1B cells.	[60]
	Inhibits proliferation of HeLa, PANC-1, and MDA-MB-231 cells and suppresses the clonogenicity of HeLa cells.	[61]
	Inhibits proliferation of HCT116 and HCCSCs cells independent of p53 status.	[62]
	Inhibits colony formation and viability of breast cancer MCF-7 cells with wild type p53, which was more sensitive	[56]
	Induces cytotoxicity in Shionogi 115 and MCF-7 breast cancer cells and PC-3 and DU-145 prostate cancer cells.	[63]
	Oral administration at 50–100 mg/kg dose suppresses growth of Capan-2 pancreatic tumor-xenograft.	[57]
	Inhibits xenograft growth and metastasis of transplanted gastric carcinoma cells in vivo zebrafish xenograft model.	[58]
	Oral feeding to mice at 40 mg/kg inhibits barcl-95 tumor growth transplanted in nude mice.	[55]
Immunomodulatory	Stimulates neutrophil functions in the immunized rats and prevents stress-induced suppression in the neutrophil functions.	[64]
	Prevents the noise-stress induced changes in cell-mediated immune response in rats.	[53]
	Ameliorates functional and histological ovalbumin-induced bronchial hyperreactivity and increases CD4 counts in lung and spleen.	[65]
	Increases cytotoxic T cells and natural killer cells in healthy human volunteers.	[66]

CAT: Catalase, SOD: Superoxide dismutase, GPx: Glutathione peroxidase, GST: Glutathione-S-Transferase, GSH: Glutathione, ROS: Reactive oxygen species, DPPH: 2,2-diphenyl-1-picrylhydrazyl, DMH: Dimethylhydrazine, B(a)P: Benzo(a)pyrene, HCCSCs: Human colon cancer stem cells. MTX: Methotrexate, LPO: Lipid peroxidation.
